# On the potential of microwave heating to convert waste into added-value chemicals and materials: a review

**DOI:** 10.1098/rsta.2024.0071

**Published:** 2025-05-22

**Authors:** Emmanuel Dan, Alan J. McCue, Davide Dionisi, Claudia Fernández Martín

**Affiliations:** ^1^ School of Engineering, University of Aberdeen, Aberdeen (ABZ), Scotland, UK; ^2^ School of Natural and Computing Sciences, University of Aberdeen, Aberdeen (ABZ), Scotland, UK

**Keywords:** microwave heating, carbon capture, sustainability, regeneration, plastic, biomass

## Abstract

Microwave (MW) heating represents a superior alternative to conventional heating techniques due to its unique ability for rapid, selective, uniform and volumetric heating. However, challenges such as temperature non-uniformity, especially in certain materials and processing conditions, can limit its widespread application. Nevertheless, this heating method can enhance the physicochemical properties and performance of materials produced, making it a vital tool in sustainable material processing to produce valuable porous carbons for CO_2_ capture, essential for climate change mitigation through carbon capture, utilization and storage (CCUS) strategies. MW heating significantly reduces processing time, energy consumption and operational costs, providing an efficient, green and cost-effective processing option. In this review we explore the use of MW heating in converting biomass, plastics and materials such as tyres and electronic waste into biofuels, bioenergy and hydrogen through biological and thermochemical processes. We highlight MW heating for producing solid adsorbents such as activated carbons from waste, their role in carbon capture and regeneration after CO₂ exposure. We also examine the principles of MW heating, its unique processing advantages and diverse applications across fields. In addition, we emphasize the life cycle assessment (LCA) of MW-assisted treatment for biomass and plastics while addressing the limitations of MW processing in material applications.

This article is part of the discussion meeting issue ‘Microwave science in sustainability’.

## 1. Introduction

Anthropogenic activities such as the burning of fossil fuels, industrialization, intensive agricultural practices and the production and disposal of plastic waste have significantly affected the environment negatively. These activities lead to climate change, pollution, loss of biodiversity and other harmful effects. To mitigate these issues, various strategies have been adopted, including the thermochemical conversion of waste into valuable products such as activated carbons and useful chemicals, which show promise in effective waste management strategies. However, traditional waste conversion processes rely on conventional heating in furnaces, which is slow, inefficient and increases energy consumption and carbon emissions due to its reliance on conduction, convection and radiation [1].

Microwave (MW) heating, leveraging the distinct properties of electromagnetic radiation, provides an energy-efficient alternative to traditional heating methods, enabling rapid and volumetric heating [2]. This can significantly reduce the carbon footprint of industrial processes. Its unique capabilities have led to widespread applications across various sectors including food processing [3], medical and pharmaceuticals [4,5], and electronics [6], due to its ability to efficiently process materials, conserve energy and reduce processing times and costs [7]. In addition, MW heating offers a sustainable solution for managing challenging waste types such as biomass, plastics, tyres, and electronic waste by converting them into valuable products [8–10]. This method stands out for its ability to penetrate materials and selectively heat specific sections of a complex waste stream without causing damage to unheated portions, provided that the targeted sections possess dielectric properties higher than those of the unheated portions. The method is adaptable in terms of power, frequency and duration adjustments, offering benefits unattainable with conventional heating techniques.

In the context of carbon capture, utilization and storage (CCUS), MW heating enhances the production and regeneration of CO_2_ adsorbents such as activated carbons and zeolites. It can yield materials with better textural properties such as better surface area and porosity [11] leading to enhanced CO_2_ adsorption performance [12], making it an integral part of sustainable material processing and CO_2_ capture strategies. In addition, the regeneration of these adsorbents through MW heating is faster [13] and more energy-efficient compared to traditional methods [14], lowering operational costs and energy consumption. This review highlights the application of MW heating to process waste materials such as biomass and plastics to useful products including activated carbons and its application in CO_2_ capture and regeneration, underscoring its role in advancing sustainable and efficient industrial practices.

### (a) MW versus traditional heating

The key differences between MW and traditional (also referred to as conventional) heating methods significantly affect their applications in chemistry, food processing and other areas. Conventional heating works by heating materials through conduction, convection and radiation, starting externally from a heat source such as an electric furnace with heat gradually moving inward, often resulting in an uneven temperature distribution [1]. This process is relatively slow and non-targeted, relying on continuous heat application and a sufficient temperature gradient to gradually distribute heat throughout the sample. In contrast, MW heating operates through dielectric heating, where materials absorb electromagnetic energy, converting it to heat from the inside out. Upon activating the MWs, the electromagnetic waves interact with the material, the oscillating electric field of the MWs cause the electric charges in the material to vibrate. This field causes the dipoles in polar molecules to continuously realign themselves with the changing electric field. Heat is generated since the molecule opposes this fast motion due to frictional forces occasioned by the continuous reorientation of the dipoles [15]. MWs can generate heat in materials with free ions or charge carriers (ionic conduction) and through interfacial polarization (heterogenous materials) or loosely attracted electrons (non-polar molecules), which, under an electric field can be shifted to create a dipole moment. This process results in fast, selective and uniform heating throughout the material. A material’s MW heating efficiency depends on its dielectric properties, including its ability to store MWs (dielectric constant, *ε*′) and convert them to heat (dielectric loss factor, *ε*″), with the heating effectiveness determined by the loss tangent (tan *δ*), which is the ratio of the dielectric loss factor to the dielectric constant, given by equation (1.1):


(1.1) $$\tan\delta \;=\;\frac{dielectric\;loss\;factor\;\left(\varepsilon^{{\;}^{\prime \prime }}\right)}{dielectric\;constant\;\left(\varepsilon^{\prime }\right)}.$$

From the loss tangent (tan *δ*), speed of light (*c*), permittivity of free space (ε_0_), the frequency (*f*), amplitude of the electric field (*E*_0_) and dielectric constant (*X*), the depth of MW penetration (*D*) and the power absorbed by the sample (*ρ*) can be deduced using equations (1.2) and (1.3) [16]:


(1.2) $$D=\;\frac{c}{2\pi f\;\sqrt{2X}\;{\left(\sqrt{1+{tan}^{2}}\left(\delta \right)-1\;\right)}^{1/2}},$$

(1.3) $$\rho =2\pi f\varepsilon_{0}\varepsilon_{0^{\;}}^{{\;}^{\prime \prime }}E_{0}^{2}.$$

**Figure 1.**
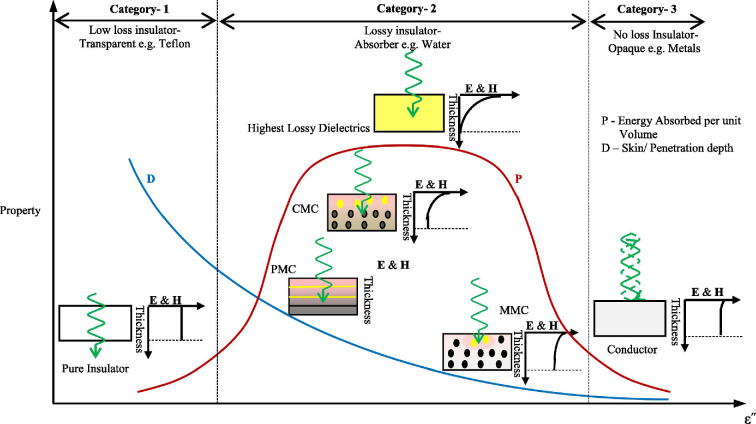
Schematic representation of MW interaction with materials of different loss tangent [22].

Materials are categorized based on their MW-absorption capacity as high (tan *δ* > 0.5), medium (0.1 ≤ tan *δ* ≤ 0.5) or low (tan *δ* < 0.1) MW-absorbing [17,18]. High-absorbing materials, such as water, silicon carbide (SiC) and graphite, efficiently absorb and convert MWs into heat. Low-absorbing materials are transparent to MWs, hence MWs can travel through them with little absorption, i.e. glass and plastics. Medium-absorbing materials such as ceramics partially transmit and partially absorb MWs. MWs are reflected by metals and cannot pass through [19] with tan *δ* >> 1 [20]. Further details on transparent, absorbing and reflecting materials, including their penetration depth and loss tangent values, can be found in the work of Bhattacharya & Basak [21]. The interaction of MWs with materials of different dielectric properties: loss tangents (tan *δ*) such as low-loss, lossy and no-loss insulators are shown schematically in figure 1.

The preference for MW heating over traditional methods in material processing is attributed to several advantages: (i) energy efficient and rapid heating significantly reduce processing times, enhancing industrial process efficiency [7]; (ii) a well-controlled heating process ensures consistent material properties [23]; (iii) selective heating allows specific reactions within mixtures to occur without affecting the entire substance, ideal for complex processing that requires varied treatment conditions [24]; (iv) lower processing temperatures save energy, especially beneficial for temperature-sensitive materials [16]; (v) improved microstructures and product quality result from MW-material interactions; (vi) MW processing is considered a green technology, reducing CO_2_ emissions and offering cost-effective benefits in time and energy savings, making it an attractive option for businesses aiming to enhance their sustainability [25].

### (b) MW applications

MW heating is increasingly preferred in various applications for its advantages over traditional heating, including sustainability and eco-friendliness (low carbon footprint). Its ability to finely control power, duration, frequency and temperature, allows for process optimization and high-quality products. However, achieving precise control without an effective feedback loop can be challenging [26]. Again, this would rely on accurate temperature measurement which is not trivial in MW application [27]. Customizable MW reactions enhance versatility, boosting its popularity across numerous sectors. Significant fields utilizing MW heating, along with specific applications, are listed in electronic supplementary material, table S1, with further discussion available in other review articles [4,6,16,28–30]. Electronic supplementary material, table S1 lists MWs being beneficial across various sectors; however, challenges remain in managing lignocellulosic waste (i.e. biomass), plastics and hazardous wastes especially when considering sustainability and environmental protection goals. Transforming these wastes into valuable and sustainable resources such as adsorbents is crucial. Using MWs to produce adsorbents from waste efficiently utilizes biomass and reduces energy use compared to traditional methods, thus lowering carbon emissions, while improving adsorbent quality and aiding in waste reduction and pollution mitigation.

MW-derived biomass and plastic adsorbents exhibit better textural properties and higher pollutant uptake capacities compared to those produced conventionally. Studies have shown that MW-produced adsorbents from materials such as cotton [31], lotus and coconut shell [32], rice husk [33], sawdust pellets [34] and plastics [9] possess greater surface areas and pore volumes. These materials also demonstrate enhanced adsorption performance with improved regeneration capabilities for various pollutants, including dyes, CO_2_ and heavy metals [13,35–37]. The use of MW technology in adsorbent production has gained significant interest, with research exploring new methods for both synthesis [12] and regeneration [13]. Beyond adsorbent production, MW techniques are applied in biomass pre-treatment and waste valorization, including plastics for hydrogen production [38] and the management of hazardous wastes, highlighting their wide-ranging effect on waste management and resource recovery.

## 2. MWs for materials processing: the fundamentals

**Figure 2.**
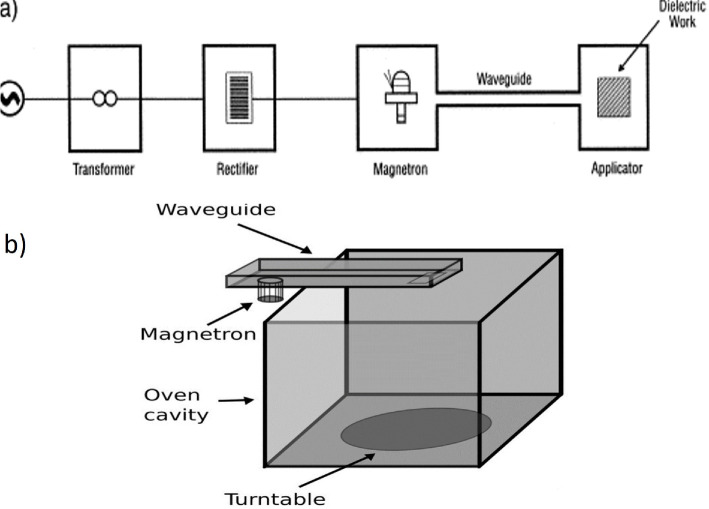
(a) MW heating system [42] and (b) MW instruments with location of components.


MW heating, using MW radiation for material processing, operates in the 0.3–3.00 GHz frequency range, with common industrial frequencies at 0.915 and 2.45 GHz, though applications outside this range exist [29,39]. MWs are generated by a magnetron and transmitted to the applicator and are either absorbed or reflected by the material being processed. Other MW sources, such as solid-state devices (e.g. transistors and amplifiers) and electron tubes, are gaining preference. Solid-state devices offer easy control over MW properties, including frequency and phase of the emitted power [40], while electron tubes remain efficient and effective at higher temperatures [41]. A diagram of a typical MW heating system, detailing component locations, is illustrated in figure 2. The MW heating effect on a material involves changes in magnetic (*H*) and electric (*E*) field strengths, governed by Maxwell’s equations. These equations, which describe electric and magnetic field interactions with matter, incorporate Gauss’s laws of electricity and magnetism, Faraday’s law of induction and Ampere’s electromagnetic law. Developed by Maxwell in 1862 [43] and subsequently revised, these laws can be specifically formulated under appropriate boundary conditions as represented in the following equations [39].

(2.1) ∇×E= ∂B/∂t,            ∇⋅B=0,

(2.2) ∇×H= ∂D/∂t+I,       ∇⋅D= ρ.

In the context provided, *ρ* represents charge density and *E*, *H*, *D*, *B* and *I* are vectors for electric field, magnetic field, electric flux density, magnetic flux density and current density, respectively. MW heating is the result of the interaction between the material and the electromagnetic field. If the material’s temperature is increased after being irradiated with MWs, it is indicative of MW absorbance and an ability to transform the absorbed energy into heat, based on the interaction that has taken place. As already stated, the extent of the response of a material to MWs is described by its dielectric constant, *ε'*, which is indicative of the capacity to store MW energy, and the dielectric loss factor, *ε″,* which indicates the ability to convert the stored energy into heat These two parameters are related as shown in equation (2.3), where j is the complex $\sqrt{-1}$ and $\varepsilon^{*}$ is the MW energy storage capacity of the material.


(2.3) $$\varepsilon^{*\;}=\;\varepsilon^{\prime }-j\varepsilon^{\prime \prime }.$$

Materials respond to electric fields in various ways, including ionic conduction, electronic, atomic, dipole and interfacial polarizations, that affect their overall dielectric properties [39]. Ionic conduction involves ions moving in response to the MW electric field, causing collisions that produce heat [44]. Electronic polarization shifts an atom’s electron cloud away from its nucleus due to an external field. Atomic and dipole polarizations occur when atoms are displaced and molecular charges align, affecting the material’s permittivity (*ε*′ and *ε*″), leading to specific responses at certain frequencies. Material interactions with MW radiation vary based on several factors: (i) frequency influences heat generation by polar molecule reorientation, with lower limits allowing reorientation and upper limits overwhelming it [45]; (ii) moisture content accelerates MW heating [46]; (iii) at higher temperatures, molecules move more freely, affecting real (*ε*′) and imaginary (*ε*″) permittivity differently depending on the frequency relation to the relaxation frequency [47]. This behaviour occurs because the dielectric absorption of polar materials is influenced by increased molecular kinetic energy at elevated temperatures. A shorter relaxation duration at high temperatures allows dipoles to align better with the MW electric field at specific frequencies, potentially increasing dielectric loss. However, if the temperature becomes too high, thermal stress may hinder alignment, reducing absorption. Interestingly, some organic acids, such as ethanoic and butanoic acids, exhibit increasing dielectric constants with rising temperatures [48]. While dielectric absorption generally decreases with temperature for most materials, this relationship is not universally consistent.

### (a) Peculiarities in MW processing of materials

MW heating is effective when materials absorb MW radiation and convert it into thermal energy. Unlike conventional heating, MW heating requires materials with adequate dielectric properties to absorb and convert electromagnetic radiation into heat. Lignocellulosic biomasses and similar materials typically have low dielectric properties, with loss tangents ranging from 0.01 to 0.07, indicating a limited ability to convert MWs to thermal energy. The dielectric properties of various biomasses, carbonaceous materials, SiC and water are listed in electronic supplementary material, table S2. However, carbonizing biomass into a biochar or activated carbon enhances its dielectric properties due to increased carbon content and aromatic structures. This process converts alkane sp^3^ carbons into alkene-like sp^2^ carbons, forming a graphitic structure in biochar that facilitates the free movement of π-electrons, leading to heating through interfacial polarization [49]. In addition, a biochar’s improved porosity, surface functional groups and lower water content, further enhance its dielectric properties [50,51].

**Figure 3.**
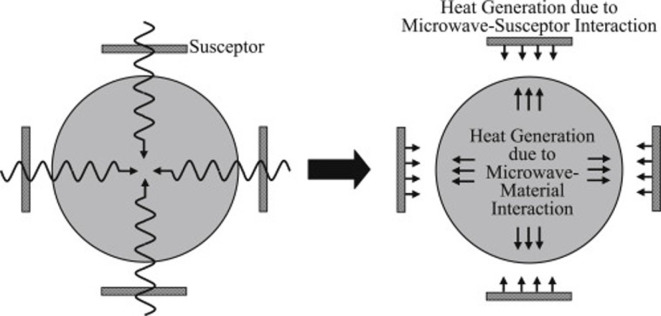
Susceptor-based hybrid MW heating in two directions [21].

MW treatment of biomass generally utilizes a MW susceptor to absorb MW radiation effectively. The susceptor is mixed with biomass to allow uniform heating without altering its chemical composition and this is known as ‘susceptor-assisted’ MW heating or ‘hybrid heating’ [21]. The process heats the susceptor first, which then transfers the heat to the biomass through conventional means until it reaches a temperature where it can directly absorb MWs for further heating. This method overcomes the uneven heating issue of direct MW heating, providing uniform heating through a combination of surface heating by the susceptor and internal heating by MWs as illustrated in figure 3.

Two hybrid heating methods for combining biomass with a susceptor have been documented—pre-mixed and non-premixed. In the pre-mixed method, biomass and susceptor are mixed before MW exposure, requiring careful blending to ensure uniform heating. The susceptor’s physical form, whether powdered, pelletized or granulated, influences the heating efficiency, suggesting matching the forms of biomass and susceptor for optimal results. This method has been used in MW pyrolysis of various biomasses such as sawdust and switchgrass [52]. The non-premixed method heats the susceptor first, then biomass is gradually introduced, allowing faster heating, but the method risks under-pyrolysis if the biomass is added too quickly. Despite these operational differences, both methods have shown similar product yields, indicating no significant advantage of one method versus the other [53]. Common MW susceptors include water, carbon black, graphite powder, charcoal and chemical substances such as SiC, aluminium, iron, iron oxide (Fe_3_O_4_ and Fe_2_O_3_), copper oxide, manganese oxide and calcium oxide [21]. These susceptors can rapidly be heated to high temperatures when exposed to MW radiation.

### (b) MW-assisted pre-treatment of biomass for its conversion to biofuels and bioenergy

**Figure 4.**
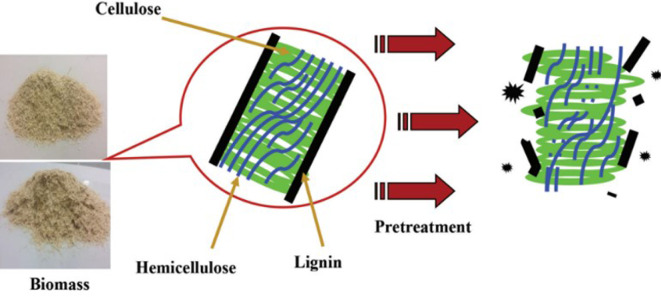
Effect of pre-treatment on lignin-bound biomass [54].

Pre-treatment involves processing biomass into valuable chemicals or energy sources, making it more accessible to enzymes or other chemicals for subsequent conversion. The process breaks down biomass into its components (cellulose, hemicellulose and lignin), as illustrated in figure 4 [54]. Pre-treatment processes for biomass, aimed at producing various biofuels and bioenergy, include physical (grinding, milling), chemical (using acids, bases), thermal (heat) and biological methods (enzymes, microorganisms), each chosen based on the biomass type and desired product. These processes are crucial for enhancing product yield, efficiency and cost-effectiveness. Emerging thermal pre-treatment technologies reported include hydrothermal, steam explosion, subcritical water hydrolysis, ultrasound-assisted processing, extrusion [55] and MW-based pre-treatment. The latter pre-treatment offers a sustainable alternative to traditional methods by reducing energy requirements and enabling selective biomass processing through MW thermal and non-thermal effects, thus accelerating various pre-treatment processes [17,56].

#### (i) MW-assisted biochemical pre-treatment for enhanced bioethanol and biogas production via fermentation and anaerobic digestion

Bioethanol production from first-generation biomass such as corn, wheat, sugarcane and soybeans involves a pre-treatment and enzyme hydrolysis process to convert polysaccharides into glucose [57]. While effective, relying on food crops for bioethanol risks global food crises and insecurity [58]. Furthermore, bioethanol yields from these crops are lower compared to those from second-generation (lignocellulosic non-food crops) and third-generation biomass (micro- and macroalgae) [57]. The abundance of lignocellulosic waste has shifted focus towards using these materials as alternative feedstocks for bioethanol production. However, challenges arise due to their complex composition, making depolymerization and enzyme digestion more difficult than with food crops [17].

The recovery of biochemicals such as bioethanol has been reported from first-generation biomass (food crops such as corn, wheat, sugarcane and soya beans) through fermentation [57]. A typical bioethanol production from food crops involves a pre-chemical treatment step before the administration of enzymes to hydrolyse the formed polysaccharides into glucose. Although the use of food crops to produce bioethanol has been quite successful, reports exist suggesting that the continued exploitation of food crops for this process might instigate food crises and insecurity worldwide [58]. This scenario and the huge availability of lignocellulosic non-food crops compared to first-generation crops have resulted in the use of these wastes as an alternative feedstock to produce bioethanol [17]. However, bioethanol production from lignocellulosic waste offers some challenges arising from its recalcitrant composition (cell walls comprise cellulose, hemicellulose and lignin) that make depolymerization and subsequent enzyme attack difficult when compared to first-generation crops [17].

MW pre-treatment of lignocellulosic materials is effective for enhancing enzyme hydrolysis by breaking down the cell wall structure and lignin-polysaccharide linkages. This process creates hotspots that cause swelling and fragmentation, making the biomass more accessible to enzymes for bioconversion to bioethanol [17,59]. MW processing enhances the efficiency of delignification and hydrolysis compared to traditional methods, requiring less chemical base and shorter times. Studies have shown that MW treatment can release up to 90% of lignin, compared to 70–80% with conventional methods over 16 h [60], and significantly speed up starch hydrolysis to glucose, requiring less acid and reducing processing time [61]. Research by Simonetti *et al.* [62] explored how MW pre-treatment affects anaerobic fermentation of food waste into bioethanol and short-chain organic acids, testing at temperatures of 120, 150 and 180°C for durations of 2, 5 and 8 min. MW treatment led to a significant reduction in volatile suspended solids, enhancing the solubilization of organic matter as indicated by increased soluble chemical oxygen demand and soluble carbohydrates, with the greatest volatile suspended solid reduction being 20%. Fermentation tests showed an increase in total product yield to 17.5% chemical oxygen demand from 11.1% for untreated substrate, indicating MW pre-treatment enhances the anaerobic digestion process for bioethanol and bio-acid production.

Studies have shown that MW pre-treatment significantly enhances bioethanol production from various biomass sources by improving the breakdown of cell wall components and increasing sugar yields. Kolo *et al.* [63] demonstrated that MW-assisted acid hydrolysis of elephant grass after alkaline delignification resulted in 47.4% cellulose, 24.3% hemicellulose and a hydrolysis efficiency of 66.57%, with sugar levels increased by 45.05% compared with conventional methods. Hu & Wen [64] achieved a sugar yield of 58.7% from switchgrass, reaching 99% of its potential sugar content using MW-assisted pre-treatment with sodium hydroxide. Chen *et al.* [65] extracted 11.9 g l^−1^ of sugars from sugarcane bagasse using MW irradiation and sulphuric acid, while Mikulski & Klosowski [66] obtained 156 mg g^−1^ of sugars from rye and wheat straw under MW conditions. Boonmanumsi *et al.* [67] reported a sugar yield of 71.64 g per 100 g (71.64 wt%) from *Miscanthus sinensis* using MW pre-treatment with ammonium hydroxide followed by phosphoric acid treatment. These findings highlight the effectiveness of MW pre-treatment in enhancing the yield of fermentable sugars from biomass, which is crucial for bioethanol production. Aguilar-Reynosa *et al.* [68] found that MW-assisted pre-treatment of maize stover led to the highest conversion rates of 95.1% to fermentable sugars and 92% to ethanol. MW-assisted pre-treatment with NaOH improved fermentable sugar production and quality, removing up to 98% of lignin from banana trunks. Yu *et al.* [69] produced bioethanol from microalgal biomass using MW-assisted wet torrefaction with dilute H_2_SO_4_. This process co-produces biochar and liquid hydrolysate, which, after fermentation with yeast, yields significant amounts of bioethanol.

MW pre-treatment significantly enhances the anaerobic digestion process of waste materials, increasing the production of biogas, CO_2_ and H_2_ from lignocellulosic feedstocks. It improves the digestibility of feedstocks and the hydrolysis stage of anaerobic digestion, leading to higher biogas yields. For instance, a study on the MW treatment of microalgae-bacterial biomass showed initial biogas production rates and final yields increase by 27−75% and 12−78%, respectively [70]. Another study employing MW pre-treatment to produce biogas from wastewater treatment found that MW irradiation at 900 W for 3 min increased methane production rates from 0.12−0.14 l CH_4_/(l∙day) to 0.16−0.20 l CH_4_/(l∙day). Damage to the microalgae cell walls was observed upon microscopic examination, thus confirming the effectiveness of MW pre-treatment [71].

Introducing sodium ions via sodium tripolyphosphate (Na_5_P_3_O_10_) to waste sludge, followed by MW treatment, effectively breaks down sludge, according to Ebenezer *et al.* [72]. This method increased solubility and reduced suspended solids by 8 and 38%, respectively, and enhanced anaerobic digestion readiness, leading to higher methane production capacity (0.615 l g^−1^) volatile solid [72]. Similarly, Serrano *et al* [73] observed that a 25 min MW pre-treatment at 700 W improved organic matter solubilization in sewage sludge, increasing both methane recovery by 16.8%, and gas production and organic loading rates by 43% and 39%, respectively. These findings recommend MW pre-treatment as a viable option for enhancing biogas production in large-scale sludge management at water treatment plants.

#### (ii) MW-assisted thermochemical pre-treatment for bioenergy production

MW-induced heating and chemical pre-treatments are used to process biomass which is characterized by high moisture, low density and low energy content, into desirable biofuels and/or bioenergy. Excessive moisture can hinder bioenergy conversion [74], making pre-treatment crucial. Biochemical pre-treatments are eco-friendly but slow, and sensitive to nutrients and contaminants, affecting enzyme functionality [75]. Thermochemical pre-treatments, favoured for their speed, flexibility and product selectivity [55], include drying, torrefaction, hydrothermal carbonization, gasification, hydrothermal liquefaction and transesterification, facilitating efficient biomass conversion to biofuels.

MW-assisted drying accelerates dehydration, enhances bio-oil yield from biomass and is 85% more energy-efficient than conventional drying methods [76]. Microwaving rice straw and sawdust pellets can produce up to 83.2% bio-oil with high energy retention, thanks to improved heating values from reduced H/C and O/C ratios [77,78]. Hydrothermal carbonization via MW heating generates hydrochar with significant surface area and micropore volume, suitable for gas adsorption without further activation. MW heating in gasification boosts syngas yield, especially enriching the H_2_ content [79]. It also aids in extracting valuable chemicals from bio-oil fractions, including alkanols and carboxylic acids. MW heating optimizes the transesterification process for biomass-derived oils, significantly enhancing the efficiency and yield of fatty acid methyl esters, achieving 99.8% yield with 99.4% purity [80]; one study showed MW heating recovered 60% of these esters in just 1 min with a MW power of 800 W, compared to only 50% recovery using conventional methods over 5 h at 60°C [81].

### (c) MW-assisted valorization of wastes

MW heating is highlighted as a unique, green and efficient alternative to conventional heating, with a reduced carbon footprint and enhanced effectiveness in waste treatment. The capability of this method to convert various waste types into valuable products through MW pyrolysis is drawing increased attention from researchers. Discussion of the MW-assisted pyrolysis of some specific waste streams is provided in the following sections.

#### (i) Biomass

Biomass primarily consists of organic materials from plant and animal sources [82], offering a sustainable alternative to fossil fuels for energy and chemical production [83]. Through processes that are widely used in various industries, biomass has the potential to reduce fossil fuel emissions and contribute to a net-zero carbon future. Produced from atmospheric CO₂, water and sunlight via photosynthesis [84], biomass materials are not only abundant and accessible but also possess favourable chemical properties, making them a valuable renewable resource.

**Figure 5.**
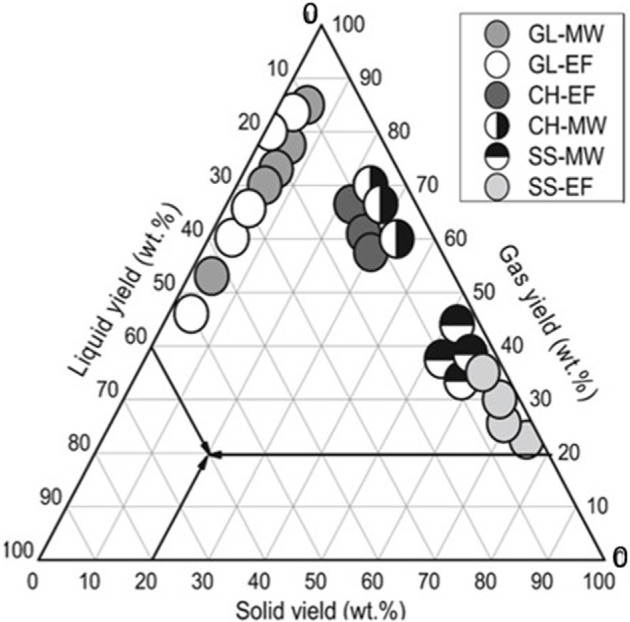
Product distributions during MW and conventional pyrolysis (EF) of glycerol (GL), coffee hull (CH) and sewage sludge (SS) [90].

Pyrolysis is a process that breaks down materials in an oxygen-free environment into solid (char), liquid (oil) and gas products, using conventional heating in a reactor influenced by temperature, time and heating rate [85]. MW pyrolysis, although similar in principle, differs in its use of MW heating and often includes MW susceptors such as SiC, graphite or activated carbon to improve MW absorption by the biomass. This method typically improves the yields and quality of products compared to conventional pyrolysis, thanks to better control over MW power, duration and the unique energy-transfer mechanism that includes micro plasma hotspots for intense and localized heating. In MW pyrolysis for biomass conversion, the liquid output generally exceeds the gas and solid yields. A study by Huang *et al.* [86] on various biomass types showed solid (biochar) yields of 16−22%, liquid (oils) yields of 40−48% and gas yields of 30−40% at 500 W. Exceptionally high liquid yields of up to 75% and 81% were reported from the pyrolysis of pinewood and cooking oil, attributed to rapid heating and minimized secondary reactions [87,88]. The liquid phase is primarily composed of oxygenated compounds, such as carboxylic acids and phenols, suggesting that the quality of bio-oil could be improved. Gas outputs, often surpass solids yields and consist mainly of H_2_, CH_4_, CO and CO_2_, with documented H_2_ concentrations of up to 18−25% [89]. The highest biochar yield noted was 60%, with a surface area of up to 800 m² g^−1^, suggesting its use as a gas adsorbent may be feasible without further activation [89]. A comparison between MW and conventional pyrolysis of some biomasses highlights the efficiency of MW methods in biomass conversion (figure 5). MW pyrolysis efficiently converts biomass into renewable energy sources such as bio-oil and syngas, suitable for heating or refining into transportation fuels. For example, it produces diesel-range bio-oil from rapeseed oil and waste cooking oil, with characteristics akin to fossil-based diesel [91]. It also yields high percentages of syngas from rice husk and microalgae. The process generates bio-oils rich in valuable chemicals (chemicals with improved values because of their distinct properties and applications).

Platform chemicals such as levulinic acid [92], furfural [93] and 5-hydroxymethylfurfural (HMF) [94]; organic acids such as acetic acid [95]; alcohols and polyols such as sorbitol [96]; specialty chemicals such as xylitol; and aromatics, including phenol [97] and benzene, toluene and xylene (BTX) compounds (benzene, toluene and xylene) [98] have been recovered from MW treatment of various biomass. These chemicals are major precursors, intermediate or end-products of various chemical and industrial processes. Levulinic acid is a key chemical in food, medicine and cosmetic industries. In addition, it is also used as a precursor to synthesize a plethora of other useful chemicals. For instance, Ƴ-valerolactone [99], 2-methyltetrahydrofuran [100], 1,4-pentanediol [101] are produced from the hydrogenation of levulinic acid and succinic acid and maleic anhydride by oxidation of levulinic acid [102]. Furfural, xylitol and lactic acid are starting materials to produce different furan-based chemicals that are utilized in food and pharmaceutical industries [103]. Among the various waste streams, lignocellulosic-based wastes such as agricultural and forestry residues, fruit peels, algal and sewage sludge represent the best biomass stream for the recovery of added-value chemicals through MW treatment. This is because this waste stream contains elevated amounts of cellulose, hemicellulose and lignin [84].

Most added-value chemicals are products of reactions involving cellulose, hemicellulose and lignin. Ethanol, levulinic acid and HMF are obtained from glucose produced through the breaking down of cellulose. Furfural is obtained from hemicellulose, while lignin is an aromatic-rich fraction that serves as a source to produce BTX phenols. The recovery of added-value chemicals through MW treatment of biomass offers several benefits compared to conventional petrochemical sources because it supports sustainability, reduces dependency on fossil fuels and represents a more economically viable option. Note that, the greatest limitation of lignocellulosic-based waste to produce added-value chemicals is the recalcitrant nature since they resist degradation either with enzymes or chemicals [82]; however, this limitation has been overcome using MW treatment techniques as previously highlighted.

#### (ii) Plastics

MW pyrolysis has been effectively used to treat plastics, first reported in 2001 [104], converting them into solid, liquid and gas fractions. This process is influenced by the type of plastic and pyrolysis conditions, such as temperature and duration. Polyolefin plastics such as polyethylene and polypropylene primarily yield high percentages of liquid products. Notable studies have reported liquid yields of up to 96% for polypropylene [105] and 86.5% for polystyrene [106], demonstrating the ability of MW heating to enhance oil yields. Outputs such as naphtha and gasoline [107] have been derived from the MW pyrolysis of plastics, including mixed plastics, showcasing the method’s versatility in generating valuable chemical resources from waste plastics.

**Figure 6.**
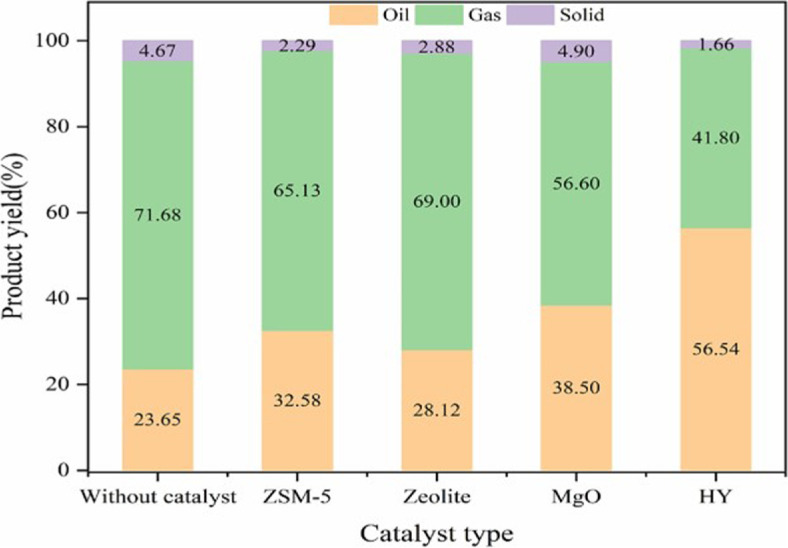
Product distribution during catalytic MW pyrolysis of LDPE over different catalyst [108].

Catalytic MW pyrolysis enhances the yield and quality of valuable products from plastics while using less energy [108]. By combining MWs with specific catalysts such as NiO and HY zeolite, the process increases the production of aromatic compounds, improving the quality of oil derived from low-density polyethylene (LDPE) by increasing its octane number [109]. It is noted that within this context, the role of the catalyst is to direct/control product selectivity for reactions occurring during MW heating; in some cases, the catalyst may also be a MW absorber and therefore influence the MW heating process. Various catalysts, such as zeolites, magnesium oxide and activated carbon, have been used to further refine the process, resulting in a significant increase in aromatic compounds in the liquid product and reducing the yield of gaseous by-products [109]. This method shifts product distribution towards more valuable liquid outputs (figure 6).

MW pyrolysis of plastics produce solid and gas fractions in addition to liquids, with solids being minimal due to low fixed carbon content in polyolefin plastics. The gas fraction contains CO, CH_4_ and H_2_, including gases up to C_3_ [110]. Notably, using catalysts such as Fe/Ni [111] and Fe–Co–Al [112] , high yields of pure hydrogen were generated from mixed plastics and LDPE, reaching up to 50.2 mmol H_2_ g^-1^ plastic [111], and 61.39 mmol H_2_ g^-1^ plastic [112]. Jie *et al.* [38] obtained a total hydrogen yield of 55.6 mmol g^−1^ plastic during MW pyrolysis of mixed plastics in the presence of FeAlO*x*. These results demonstrate MW pyrolysis as an effective and sustainable method for converting plastic waste into valuable hydrogen, supporting the circular economy and potentially reducing future reliance on fossil fuels, thereby lowering CO_2_ emissions towards achieving NetZero by 2050 goals (certainly relative to conventional pyrolysis of waste plastic). Stubborn waste types such as plastic tyres, waste electrical and electronic equipment and medical/pharmaceutical wastes have all been effectively managed using MWs to obtain useful products including H_2_ and CH_4_ and other functional chemicals. The reader is directed to other reviews for further details [10,113–115].

## 3. MW-assisted synthesis of porous solid materials: applications

MW heating is known for its ability to enhance the properties and performance of materials by allowing energy to penetrate and heat the entire volume simultaneously, leading to rapid and efficient heating. This process, documented by Klinowski *et al.* [11] and others, operates at lower temperatures and requires less energy [9] than conventional methods, making it an effective alternative for synthesizing a variety of materials, including biochar, activated carbons, graphene, carbon nanotubes, zeolites, metal organic frameworks (MOFs) and covalent organic frameworks (COFs). The detailed benefits of MW heating on the synthesis of these materials are listed in table 1.

**Table 1.** MW effect on some porous materials processing

porous material	effect of MW heating during production	references
activated carbons	(i) enhances pore structure development, (ii) shorter activation time, lower processing temperatures, hence energy saving (iii) higher adsorption capacity	[35,116]
zeolite	(i) shorter reaction time, (ii) results in uniform crystallinity	[117]
graphene	(i) accelerate the reduction rate of graphene oxide to graphene	[118]
carbon nanotube	(i) short process time, (ii) improves kinetic parameters	[119]
MOFs	(i) reduces the time of reaction (ii) improves phase selectivity, (iii) provides control over crystal morphology	[120] [121]
COFs	(i) increases process reproducibility, (ii) results in the isolation of a cleaner/purer product	[122]

Activated carbons (ACs) are low-cost and sustainable adsorbents produced from widely accessible and cheap carbon precursors such as biomass [123] and plastics [9]. In addition, ACs are preferred over other adsorbents due to their effectiveness in the remediation of a wide range of pollutants, including dyes, trace metals, organics [124,125] and gases [34,126], and are often characterized by low-regeneration energy requirements relative to other competing technologies. Typically, ACs are produced by the carbonization of the precursor to obtain char and the subsequent activation of that char by physical or chemical methods. The carbonization and activation stages utilize heat either from conventional sources or MWs; however, the former has been criticized for being slow, requiring high energy input and thus resulting in an increase in cost during AC production. MW heating offers a more sustainable and cost-effective alternative, utilizing the fast and uniform heating mechanism to produce AC with less energy consumption. The use of MW heating can also enhance textural and chemical properties and overall material performance beyond what is achieved by traditional heating methods [8,9,33]. MW heating can also result in ACs with improved yield and greater thermal stability [31].

As previously stated, carbonaceous wastes such as biomass and plastics are plentiful precursors for AC production. The application of MWs to convert these wastes into high-performance CO_2_ adsorbents has already been demonstrated. Electronic supplementary material, table S3 lists the CO_2_ uptake performance of selected ACs produced from biomass and plastics by MW treatments as well as values for zeolites, MOFs and COFs. The results indicate that MW heating produce ACs with varying CO_2_ adsorption capacities, influenced by both the carbon source and preparation conditions used. MW heating is effective in creating micropores within materials, which enhance CO_2_ adsorption. The process typically takes less than 15 min, with optimal MW activation times under 6 min leading to ACs with substantial adsorption capacities [9], though a few required more time [8]. The temperatures used in MW activation are lower (not more than 400°C) than the 700–1100°C typically required in traditional activation methods, offering a more efficient way to maximize performance [127]. The short activation time and low activation temperature all have a positive effect on the cost of AC production [9].

Notably, MW heating of biomass can directly produce ACs with significant adsorption capacities without needing further activation [128]. This is partly due to the development of micro plasma spots at low temperatures, which help create pores and increase the materials surface area [86]. Furthermore, MW heating under a nitrogen atmosphere can lead to N being incorporated into the AC structure, increasing surface basicity and subsequently the CO_2_ adsorption capacity [8]. This N incorporation bypasses the need for conventional doping methods, simplifying the synthesis process. However, some MW-ACs have lower adsorption capacities compared to other porous solid adsorbents. Despite this, the MW production of adsorbents from biomass and plastics offers additional benefits, including a reduced environmental effect, reduced use of hazardous chemicals and an effective waste management strategy. This approach supports the circular economy by converting biomass and plastic wastes into valuable carbon products. Moreover, optimizing MW power, temperature and activation time might enhance the adsorption capacities, possibly surpassing those of zeolites and other porous adsorbents. Beyond the adsorption of CO_2_, MW-produced ACs also demonstrate effectiveness in the adsorption of other gases, such as NO_2_ [34], H_2_S [129], SO_2_ and CH_4_ [130,131].

### (a) MW regeneration of solid materials

Over the past 80 years, the application of MW heating in chemical processes has transitioned from a basic heating technique to a valuable process intensification method for enhancing efficiency and reducing costs across almost all chemical and process industries [132]. In the realm of adsorbent regeneration, MW heating has emerged as a superior alternative to conventional thermal methods, demonstrating greater efficiency (in terms of capacity, kinetics and energy input) for a variety of adsorbents. Early experiments successfully regenerated molecular sieves (type 4A, 13X zeolites) and silica gel loaded with tritiated water at 350°C using MWs without loss in adsorption capacity [133], and similar high efficiency was observed for activated alumina (F-200) loaded with methanol [134]. Furthermore, Cha & Kong [135] observed an increase in NO*x* adsorption capacity in coke-derived char after nine regeneration cycles. Bradshaw *et al.* [136] reported that MWs regenerated granulated activated carbon (GAC) pre-soaked with moisture to its full capacity, suggesting significant cost savings could be achieved in the recovery of gold according to the study. Fast desorption rates were also achieved for GAC loaded with solvents such as butanone and acetone [137], with more than 97% efficiency reported for GAC used for treating phenol and wastewater pollutants over 10 cycles [138,139]. Regenerating adsorbents efficiently undoubtedly plays a vital role in CCUS, significantly affecting the operational costs associated with the energy requirements for desorption [140]. In line with this, Zheng *et al*. [141] reported that the capture process has a penalty in efficiency between 10 and 14%, and Hong [142] indicated that the most expensive element of CCS/CCUS is the CO_2_ capture process, accounting for the 50% of the total costs of CCS. Accordingly, reducing energy consumption during desorption is crucial for lowering CO_2_ capture costs. Gomez-Rueda *et al.* [143] highlighted MW regeneration as an exceptionally fast method, achieving 100% efficiency in 80 s at 150 W while maintaining the microporosity of an AC after multiple cycles.

Yassin *et al.* [144] conducted a comparative study to assess the effects of MW versus conventional heating on regenerating two commercial adsorbents—a molecular sieve (13X) and an activated carbon (Norit R2030CO2)—both previously exposed to a binary CO_2_/N_2_ gas mix simulating pre-dried flue gas from coal-fired power plants. The study found that MW heating offered more stable adsorption capacities and regeneration efficiencies for both types of adsorbents compared to conventional heating. However, this stability in MW heating only manifested after an initial drop in capacity during the first cycle for 13X at the regeneration conditions tested. Notably, Norit R2030CO2 regenerated with MW heating showed a higher maximum desorption rate than with conventional heating, a result not observed with 13X. Overall, MW-assisted regeneration outperformed conventional heating in maintaining capture capacity and regeneration efficiency for Norit R2030CO2. A follow-up study examined heating techniques and moisture effects on commercial adsorbent regeneration using a synthetic flue gas mixture (15% CO_2_ in N_2_) dry and with a 15% relative humidity added [135]. Norit R and zeolite 13X were more efficient at adsorption (working capacity) with a moist gas feed when regenerated with MWs than with conventional methods. MW regeneration achieved desorption efficiencies of 96% for Norit R and 90% for zeolite 13X, which are higher compared to the 81.7 and 76.7% efficiencies achieved through conventional methods. This suggests that MW regeneration may be a more effective method in humid conditions due to its compatibility with water. This is attributed to the enhanced regeneration ability of MW heating.

**Figure 7.**
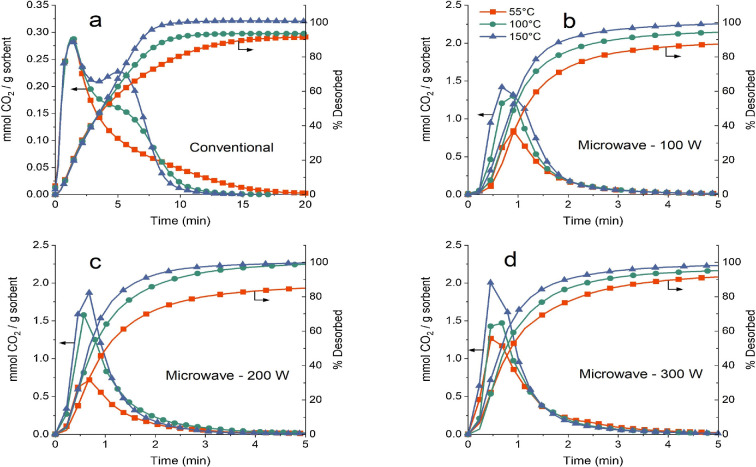
Desorption curves for CO_2_ at different temperatures: filled square (55°C), circle (100°C) and triangle (150°C) [14].

Ellison *et al.* [14] explored how temperature (50–150°C) and MW power (100–300 W) affected the regeneration of zeolite 13X, previously exposed to 15% CO_2_, comparing conventional and MW heating. The study found that while conventional heating required 10–20 min for full desorption depending on the bed temperature, MW-assisted regeneration achieved complete desorption in only 5 min (figure 7). This faster result with MWs is attributed to the more efficient and therefore faster heating method. Erguvan & Amini [145] reported that zeolite 13X, when exposed to the same CO_2_ concentration, achieved full desorption in just 8 min using a lower MW power input of 15 W. Mesoporous MCM-48 silica, modified with silane groups bearing one to three amine groups, was utilized for CO_2_ adsorption (15% concentration) at 25°C, followed by regeneration through either conventional heating or MW irradiation. The findings indicated that using a triaminosilane derivative for functionalization yielded the highest CO_2_ adsorption capacity and the quickest regeneration via MW heating, achieving a regeneration rate four times faster than the unmodified material. In addition, the modified adsorbent maintained full stability for at least 20 adsorption–regeneration cycles [146].

MW regeneration consumes less energy than conventional heating, leading to significant energy savings. Yassin *et al.* [13] found that MW regeneration of activated carbon (Norit R) was more energy-efficient than conventional methods. They found that the power consumption per adsorbent unit mass and per adsorbate removed, were reduced with MW regeneration by 18.69 and 17.76%, respectively. Yang *et al.* [147] reported a 10% reduction in energy use during MW regeneration of perfluorinated silica-stabilized dry alkanolamines compared to conventional heating. Erguvan & Amini [145] observed that fully regenerating zeolite 13X with MWs required only 3.87 MJ kg^−1^ CO_2_ at 40°C lower than the 4−6 MJ kg^−1^ CO_2_ needed for amine CO_2_ absorption [148] and significantly less than the 6−7.9 MJ kg^−1^ CO_2_ reported for conventional heating of zeolite 13X [149,150], which translates to a 35–49% reduction in energy consumption. This efficiency is attributed to MW regeneration utilizing MW energy directly, in contrast to conventional heating method that relies on electrical energy.

## 4. Life cycle assessment (LCA) of MW application in biomass and plastic waste treatment

The conversion of biomass and plastic wastes to added-value products using MW treatment requires different input materials (such as chemicals, water and gases), land and energy resources as well as outputs (air, water and solid wastes). These input materials are required for the whole process from feedstock pre-treatment to product recovery and purification. Waste materials of different forms are consequently produced all through these stages. Although MW-assisted conversion of wastes to added-value products is widely considered as a green process [151], a holistic evaluation of the environmental effect of the total conversion process would be crucial. LCA results would allow for easy comparison of MW production route with other methods and the choice of the best route based on the least environmental effects.

The LCA studies summarized within this review were conducted within a system boundary: cradle-to-gate, at 1 kg functional unit (except where otherwise stated) and exclude environmental effects associated with the later stages of processing of products and the transportation of the products generated from pyrolysis.

Parthasarathy *et al.* [152] reported that MW pyrolysis resulted in a higher global warming potential, GWP (14.94%), ozone layer depletion (14.29%) and photochemical oxidation (14.44%) than conventional pyrolysis in their LCA for the conversion of waste date stones to biofuel. Studies have explored the environmental effect of MW-assisted processes for biofuel and bio-product production from various feedstocks, showing mixed results in comparison to conventional methods [153]. They reported that MW catalytic pyrolysis of switchgrass, especially at lower temperatures (300°C), yielded a significantly lower GWP than conventional pyrolysis. The use of a K₃PO₄ catalyst reduced energy consumption and emissions, with GWP ranging between 159 and 223 kg CO₂-eq. per 1000 kg of switchgrass, making it an environmentally favourable option due to improved efficiency and carbon sequestration potential through biochar. Similarly, a study found that MW-assisted pyrolysis of pine sawdust had favourable environmental effects in most categories, though it showed notable contributions to global warming potential (1.18 kg CO₂ eq.), photochemical oxidant formation (0.71 kg NMVOC eq.) and human toxicity (2.46 kg 1,4-DCB eq.) [154]. Biomass production and non-condensable gas emissions during pyrolysis contributed significantly to these effects.

MW-assisted pre-treatment of a multi-product biorefinery reduced energy demand by 5.82% and lowered environmental effects such as global warming and fossil resource scarcity by approximately 5% compared to conventional hydrothermal methods, making it more sustainable for biorefinery applications based on the report [155]. It was also reported that MW-assisted hydrothermal carbonization of sugarcane bagasse had high energy demands, resulting in notable effects on climate change and fossil depletion [156]. In addition, liquid discharge from MW-assisted hydrothermal carbonization caused severe eutrophication effects, indicating environmental trade-offs. Another study also found that catalytic MW pyrolysis of mushroom spent compost was less environmentally favourable than composting, with significantly higher greenhouse gas emissions (fourfold), photochemical smog (twofold) and toxicity levels (tenfold) [157]. Regarding MW plastic pyrolysis, the environmental effect of the MW co-pyrolysis of food waste and LDPE into bio-oil, biochar and biogas resulted in an emission of 38.92 kg of CO₂ equivalents per 100 kg of feedstock [158]. The drying and pyrolysis units contributed 91% of the total energy consumption and were major sources of greenhouse gas emissions. Additional emissions included 0.048 kg of SO₂, 0.0077 kg of phosphate and 1.1 kg of dichlorobenzene equivalents. However, the produced bio-oil and biochar offer environmental benefits by reducing fossil fuel demand and supporting soil carbon sequestration according to the study. Another study by Muniyappan *et al*. investigated the environmental effect of MW co-pyrolysis for converting waste electrical plastic and seed cake into renewable fuel [159]. They reported 771.95 kg of CO₂ emissions per tonne of feedstock, along with 3.11 kg of SO₂ (acidification), 1.69 kg of phosphate (eutrophication), 338.59 kg of dichlorobenzene (toxicity) and 0.11 kg of ethylene (oxidant potential). Reducing electricity use by 15% could lower the effect by 13.69%. It was highlighted that MW co-pyrolysis is a promising waste-to-energy solution, though energy optimization is crucial for reducing its environmental footprint [159].

## 5. Limitation of MW-assisted biomass treatment

MW heating offers a promising alternative to conventional heating for biomass and plastic processing, yet faces several limitations. One major challenge is the heterogeneous composition of biomass feedstocks, with varying levels of cellulose, hemicellulose, lignin, extractives and ash depending on the source, making uniform processing difficult [160].

Pure biomass does not heat effectively under MWs without an external absorber (susceptor), requiring hybrid heating techniques [161–163]. This complex method heats the material both from the surface, via the MW absorber and from the centre [21], leading to uneven temperature gradients [163] and creating hot and cold spots that compromise processing efficiency and quality of product. These temperature inconsistencies can cause thermal runaway [164,165], risking equipment, especially when the susceptor’s properties vary with temperature [51].

Another issue with non-uniform MW absorption in biomass during hybrid heating is that MW heating is volumetric [166] and depends on the material volume, positioning and cavity size. When treating large volumes in a large cavity, uneven MW absorption can occur due to variations in electromagnetic fields, penetration depth and thermal diffusion, leading to non-uniform heating [167]. Various factors, such as moisture content [168], sample size [169], penetration depth [170] and cavity design [171], further influence MW absorption and temperature distribution [168], leading to inconsistencies in experimental results.

There are limited data on the dielectric properties of many biomass materials [168], which is essential for designing effective MW experiments and predicting MW absorption [172,173]. Dielectric properties are critical for understanding how materials behave under MW irradiation and assessing experimental feasibility [172]. Although some studies have investigated the dielectric properties of biomass-derived char [174,175], some of these results are inconsistent and mostly exclude raw biomass and mixtures [51]. Also, most char dielectric data are collected at room temperature using a single frequency, neglecting how dielectric properties vary with temperature, frequency and post-treatment changes.

There is limited knowledge on the stages and mechanisms of biomass transformation under MW irradiation, including the phases and intermediate structures that lead to final products [176]. Most studies focus on the end results of MW treatment, analysing the final product’s physical and chemical properties and performance compared to conventional heating methods. While some research has explored transformation mechanisms and kinetics, few experimental studies track the decomposition process in real time, and no *in situ* studies exist to observe decomposition as it happens. This gap limits understanding of the unique effects of MWs on the transformation process.

In a MW cavity, fixed patterns of high- and low-energy areas, known as hot and cold spots, arise from constructive and destructive wave interference. This uneven heating makes it difficult to measure the bulk sample temperature accurately. While temperature sensors such as thermocouples [177], infrared cameras and optical pyrometers [160] can measure specific points, each has limitations. Metal-based thermocouples can interfere with MWs, causing inaccuracies, and infrared pyrometers are unreliable for solids and struggle at temperatures above 300°C [12]. Combining methods such as thermocouples and pyrometers has been suggested to improve accuracy, but this requires a complex set-up [85].

## 6. Conclusion

MW heating is widely used across various sectors for its efficiency and cost-effectiveness, particularly in transforming waste into biofuels and enhancing the quality of activated carbons for better CO_2_ adsorption. This method is also notable for its energy-efficient and fast regeneration process. Its effectiveness in these areas makes MW heating crucial for sustainable practices and carbon capture efforts, highlighting its importance in promoting environmental sustainability. Despite its many benefits, the use of MWs in material processing still faces numerous challenges for optimization as highlighted. Beyond MW-specific effects such as plasma hotspots (non-thermal effects), there is limited understanding of the real interactions between MWs and materials and the changes that occur during heating. This knowledge gap can be addressed by integrating MWs with infrared cameras and spectroscopic tools such as Raman spectroscopy. These tools can provide detailed analyses of molecular vibrations, crystal structures and phase transitions, aiding the understanding of how materials change under different temperatures and MW power regimes. In addition, MW cavities should be designed to accommodate various configurations of sample reactors for optimal heating, rather than providing a fixed point for reactor installation. Furthermore, accurately measuring power consumption is crucial if MW technology is to be implemented in new processes, as it must demonstrate clear advantages over conventional methods.

## Data Availability

Supplementary material is available online [178].
